# Molecular Phylogenetics and Biological Potential of Fungal Endophytes From Plants of the Sundarbans Mangrove

**DOI:** 10.3389/fmicb.2020.570855

**Published:** 2020-11-13

**Authors:** Md Sohanur Rahaman, Md Afjalus Siraj, Sabiha Sultana, Veronique Seidel, Md Amirul Islam

**Affiliations:** ^1^Pharmacy Discipline, Life Science School, Khulna University, Khulna, Bangladesh; ^2^Department of Pharmaceutical Sciences, Daniel K. Inouye College of Pharmacy, University of Hawaii at Hilo, Hilo, HI, United States; ^3^Agrotechnology Discipline, Life Science School, Khulna University, Khulna, Bangladesh; ^4^Natural Products Research Laboratory, Strathclyde Institute of Pharmacy and Biomedical Sciences, University of Strathclyde, Glasgow, United Kingdom

**Keywords:** bioactivity, ecosystem, endophytic fungi, phylogenetics, fungal metabolites, symbiosis

## Abstract

The Sundarbans forest in Bangladesh is the world’s largest mangrove. It is a unique ecosystem where living organisms face extreme challenges to compete for survival. Such competition results in the production of bioactive molecules which are useful for agriculture and human health. In this study, eighty fungal endophytes from nine mangrove plants growing in a region, as yet unexplored, of the Sundarbans were isolated by surface sterilisation and pure culture techniques. Among the eighty isolates subjected to a preliminary antimicrobial screening using an agar plug diffusion assay, only fifteen showed some promising activity. These were subsequently identified by polymerase chain reaction of their ITS gene. Extracts prepared from the identified isolates were screened for antimicrobial, antioxidant, cytotoxic and α-glucosidase inhibitory activities. Their total polyphenol and flavonoid content and their FRAP value were also determined. All endophytes are reported for the first time in the plants under investigation.

## Introduction

The Sundarbans mangrove, located in the coastal region of Bangladesh, is the largest salty swamp forest in the world (Iftekhar and Saenger, 2008). It is a unique environment, at the interface between the marine and the terrestrial ecosystems, with a rich biodiversity of plants, animals and microorganisms that have evolved to adapt to specific environmental conditions such as high salinity and moisture, high temperatures, low oxygen, and periodic flooding (Gopal and Chauhan, 2006; Castro et al., 2014). Reports have showed that many plant species from tropical mangroves harbor microorganisms such as bacterial and fungal endophytes that live within the plant tissues (Bugni and Ireland, 2004; Castro et al., 2014). Such microorganisms have been reported to produce a variety of secondary metabolites to protect the host plants (Strobel et al., 2004; Gunatilaka, 2006; Arora et al., 2018). These metabolites have found many applications in agriculture and in the discovery of new drugs and include compounds with some antimicrobial activity (Schulz et al., 2002; Strobel, 2003; Schueffler and Anke, 2014). More specifically, fungal endophytes isolated from mangrove plants have displayed anticancer, antimicrobial, antiviral, antitubercular, anti-inflammatory, anti-oxidant, antidiabetic, antiparasitic, antihypertensive and immunomodulatory activity (Tejesvi et al., 2008; Proksch et al., 2010; Kaul et al., 2012; Salini, 2015; Bibi et al., 2020). Examples of biologically active metabolites produced by fungal endophytes include pestalotiopsones A-F, talaperoxides A–D, steperoxide B (Li et al., 2011), xylacinic acids A-B, cytochalasin D, isosclerone (Klaiklay et al., 2012), nectriacids A–C and 12-epicitreoisocoumarinol (Cui et al., 2016). This study was performed to investigate the diversity and biological potential of fungal endophytes isolated from nine plants, namely *Avicennia alba*, *A. marina*, *Bruguiera sexangula*, *Brownlowia tersa*, *Ceriops decandra*, *Derris trifoliata*, *Heritiera fomes*, *Xylocarpus moluccensis* and *X. granatum*, collected from the Munshiganj range (Satkhira district) of the Bangladeshi Sudarbans mangrove forest, an area as yet unexplored for the presence of endophytes. To the best of our knowledge, *Brownlowia tersa*, *Ceriops decandra* and *Derris trifoliata* have never been investigated for the presence of fungal endophytes.

## Materials and Methods

### Endophytic Fungi Isolation

**FIGURE 1:**
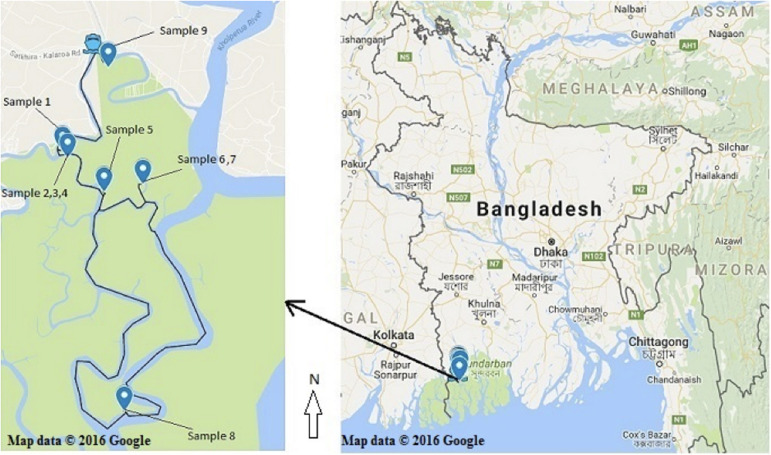
Recorded collection sites of the nine plant species from the Sundarbans mangrove. (Map courtesy: Google map; accessed date. 05/05/2016).

Healthy (without any symptom and disease) bark, stem, leaves and pneumatophores were collected in May 2016 from nine plant species found in the Satkhira district (Munshiganj range) of the Sundarbans mangrove forest in Bangladesh. Samples were collected from muddy zones of tidal salty water and each collection point was carefully documented on the map by recording its GPS coordinates (Supplementary Table 1 and Figure 1). All plant specimens were identified by one of the authors and a voucher specimen for each sample was preserved in the Plant Pathology Division (Agrotechnology Department, Khulna University, Bangladesh). The collected plant parts were processed according to a standard procedure with some slight modifications (Kjer et al., 2010). Briefly, the samples were washed thoroughly with tap water and cut into small pieces (1 cm × 1 cm) followed by surface sterilization *via* successive immersion into 75% ethanol for 1 min, 0.5% sodium hypochlorite for 3 min and finally rinsed with sterile distilled water. The samples were then placed horizontally onto petri-dishes containing Potato Dextrose Yeast Agar (PDY) supplemented with 150 mg/L chloramphenicol. The plates were incubated at 25 ± 2°C with a 12/12 h light/dark period and checked every day for fungal growth. Once a mycelium was present, the hyphal tips were removed from the plates using a sterile sharp needle and were carefully transferred to a new PDY agar plate avoiding cross-contamination. The pure isolates were obtained after several similar sub-culturing onto fresh PDY plates.

### Endophyte DNA Extraction, Amplification and Sequencing

After sufficient growth on Potato Dextrose agar (PDA) for 7–10 days, the mycelium of each endophyte was scraped from the PDA medium and stored at −80°C for further use. The stored mycelium was ground in a mortar under liquid nitrogen and approximately 35 mg was taken into a micro centrifuge tube. Then the fungal genomic DNA was extracted using a PureLink^®^ fungal DNA isolation kit (Invitrogen, United States) according to the manufacturer’s protocol. The extracted DNA was quantified by spectrophotometry and 50 ng/μL was used for each polymerase chain reaction (PCR). Fungal internal transcribed spacer (ITS) DNA, located between a small subunit of rRNA and a large subunit of rRNA gene, was amplified using ITS-5 (5′-GGAAGTAAAAGTCGTAACAAGG-3′) and ITS-4 (5′-TCCTCCGCTTATTGATATGC-3′) primers using the PCR conditions previously described (Wu et al., 2015). Each PCR mixture consisted of 50 ng DNA template, 0.16 mM dNTPs, 0.16 μM forward and reverse primers, 1.25 mM MgCl_2_, 10 × PCR buffer and 0.04 U taq polymerase. The PCR product was visualized on 1% agarose gel by electrophoresis. Each product from a successful PCR was purified using the PureLink^®^ PCR Purification Kit (Thermo Scientific, United States) and sequenced as previously described (Schoch et al., 2012).

### Phylogenetic Analysis

Each sequence was fine-tuned by removing the noise and ambiguous peaks followed by combining the forward (5′–3′) and reverse (3′–5′) sequence to generate an accurate consensus sequence, which was used for a BLAST (Basic Local Alignment Search Tool) search to retrieve the identity of the endophytic isolates from the existing INSDC (International Nucleotide Sequence Database Collaboration) databases. The INSDC sequences which showed the greatest similarity with the query sequences of our endophytic isolates were obtained and multiple sequence alignment was performed with Clustal W^®^ (Thompson et al., 2003). The appropriate model suitable for the dataset and the AIC (The Akaike information criterion) was determined from Jmodeltest^®^ v.2.1 (Guindon and Gascuel, 2003; Darriba et al., 2012). Then the phylogenetic evolutionary tree (Figure 8) was generated using PHYML^®^ v.3.1 (Guindon et al., 2010) with the optimized parameter calculated by Jmodeltest^®^ v.2.1 (Guindon and Gascuel, 2003; Darriba et al., 2012). Only a 2% sequence divergence limit was allowed to assign a taxon to species level. A query coverage of 90–100% and an expected value of 0 were assigned to allow for an uncompromised quality control of the identification.

### Reagents, Chemicals, and Instruments

All microbiological isolation and fermentation media were purchased from Himedia Bioscience (India). The primers for the polymerase chain (PCR) reactions were procured from Integrated DNA Technologies, Inc. (Coralville, United States) and other PCR reagents from Sigma-Aldrich (St. Louis, United States). Azithromycin, amoxicillin, nystatin, quercetin, gallic acid, α-glucosidase, pNPG and all the anti-oxidant reagents were purchased from Sigma-Aldrich (St. Louis, United States). Acarbose was obtained from ACME Laboratories Ltd. (Bangladesh). A Multiskan GO (Thermo Scientific) instrument was used as a microplate reader. The PCR used a T Professional (Biometra) thermal cycler. The ATCC strains used for the antimicrobial screening were obtained as Culti-loops from Remel Inc. (Thermo Fisher Scientific, San Diego, CA, United States). All solvents and chemicals used in this study were of analytical grade and of the highest purity, respectively.

### Bacterial Strains and Cancer Cell Lines

*Escherichia coli* (ATCC^®^ 8739), *Staphylococcus aureus* (ATCC^®^ 6538), *Staphylococcus aureus* (ATCC^®^ 29737), *Bacillus subtilis* (ATCC^®^ 6633), *Salmonella enterica sv typhimurium* (ATCC^®^ 14028), *Salmonella species sv Abony* (NCTC 6017), *Kocuria rhizophila* (ATCC^®^ 9341), *Candida albicans* (ATCC^®^ 10231), *Aspergillus brasiliensis* (ATCC^®^ 16404), Human Breast Adenocarcinoma (MCF-7 ATCC^®^ HTB-22TM) and Human Lung Adenocarcinoma (SK-LU-1; ATCC^®^ HTB-57TM) cell lines were obtained from the American Type Culture Collection (ATCC).

### Preliminary Screening for Biological Activity

All endophytic fungal isolates were assessed for antimicrobial activity against two Gram positive bacteria (*S. aureus* and *B. subtilis*), two Gram negative bacteria (*E. coli* and *Salmonella* species), and two fungi (*C. albicans* and *A. brasiliensis*) according to a procedure previously described (Santos et al., 2015; Balouiri et al., 2016) with some slight modifications. Briefly, each isolate was cultured onto PDY agar plates without antibiotic at 25°C for 25 days. Then, a circular agar block (7 mm) was picked from the plate using a cork borer and was placed onto Mueller-Hinton agar (MHA) plates previously inoculated with each bacterium and PDA plates previously inoculated with each fungus. The agar block was placed within 5–10 min of bacterial or fungal strain inoculation. A sterilized filter paper disk impregnated with sterile distilled water (SDW) was used as the negative control. Kanamycin (30 μg/disc) and ketoconazole (50 μg/disc) were used as positive controls for all bacterial and fungal strains, respectively. Following incubation at 37°C for 16–24 h (bacteria) and at 35°C for 48 h (fungi), antimicrobial activity was measured as the zone of inhibition (in mm) around the agar block. The assay was performed once in duplicate. Only isolates showing activity were selected for further analysis.

### Scaling Up of the Endophytic Liquid Cultures

The fermentation of endophytic fungi was carried out as previously described (Li et al., 2017). The preparation of extracts from fungal isolates was performed according to a previous methodology (Borges et al., 2011) with some modifications. After 7–10 days of incubation, a pre-inoculum of each endophytic isolate was prepared by transferring 2–3 grown isolates into an Erlenmeyer flask containing 40 mL Potato Dextrose Broth (PDB) supplemented with 0.5% yeast extract followed by incubation at 25 ± 2°C for 5–7 days with some occasional shaking (180 rpm). The final fermentation step was performed by inoculating the pre-inoculum into a 1 L Erlenmeyer flask containing 300 mL PDB supplemented with 0.5% yeast extract and incubating at 25 ± 2°C in the dark for 4–6 weeks also with some occasional shaking (180 rpm). After fermentation, the broth was filtered with Whatman filter paper no. 1 and the mycelium was discarded. The filtered broth was defatted with *n*-hexane (1:1 v/v), and then extracted with ethyl acetate (EtOAc) (1:1 v/v). Dried extracts were obtained following evaporation to dryness under reduced pressure.

### Antimicrobial Assay and Minimum Inhibitory Concentration (MIC) Determination

Minimum inhibitory concentrations (MICs) were determined for each fungal extract using a broth microdilution assay according to a methodology previously described for bacteria (Wiegand et al., 2008), yeasts (Pfaller et al., 2004) and filamentous fungi (Masoko et al., 2007) with some slight modifications. Mueller-Hinton broth (MHB), PDB and Sabouraud Dextrose Broth (SDB) were used for bacteria, yeast, and filamentous fungi, respectively. Each endophytic fungal extract was dissolved in acetone to prepare a stock solution of 6 mg/mL and 50 μL of this stock was mixed with 50 μL SDW into a 96-well plate followed by 10 times serial dilution with 50% SDW to obtain a range of concentrations from 3 to 0.005 mg/mL. Bacterial or fungal suspensions were standardized to a 0.5 McFarland standard using optical density measurements, and within 15 min of standardization were inoculated (50 μL) into the wells containing the extract as well as the growth control well (broth and microbes only). Azithromycin and amoxicillin were used as positive controls for bacteria, and nystatin was used as a control for the fungi. Other controls added to each plate included a medium-only, SDW-only and solvent-only wells. The plates were incubated either for 16–20 h at 37°C (bacteria), 24 h at 35 ± 2°C (yeast) and 46–50 h at 35 ± 2°C (filamentous fungi). Following incubation, MICs were detected by addition of 10 μL of a resazurin (Sigma-Aldrich, United States) solution (0.02% w/v) to each well and further incubation of the plate for 1–6 h. The absence of visible microbial growth was indicated by the blue color of the resazurin dye while a pink color indicated positive growth. Prior to the addition of the dye, aliquots (10 μL) were taken from the wells showing no visible growth, and were inoculated onto fresh Mueller Hinton, Potato Dextrose, and Sabouraud Dextrose agar plates for bacteria, yeast, and filamentous fungi, respectively. Following incubation for 18–24 h at 37°C (bacteria), at 35°C (yeast) or 46–50 h at 35°C (filamentous fungi), and colony counting, the minimum bactericidal concentrations (MBC) and minimum fungicidal concentrations (MFC) were defined as the lowest concentrations at which less than one colony was visible on the agar plates. If more than one colony was observed, this indicated a bacteriostatic/fungistatic activity (Pujol et al., 2000; Muzaffar et al., 2016). Each sample was screened in triplicate.

### DPPH (2, 2-Diphenyl-1-Picrylhydrazyl) and ABTS (2, 2′-Azino-Bis, 3-Ethylbenzothiazoline-6-Sulphonic Acid) Free Radical Scavenging Assays

The DPPH radical scavenging assay was done according to a previously published protocol (Cui et al., 2015) with some modifications. Endophytic fungal extracts were dissolved in methanol to prepare stock solutions of 1 mg/mL. The stock solutions were serially diluted into the 96-well plate with methanol to obtain a range of concentrations from 1,000 to 3.90 μg/mL. A 0.2 mM DPPH (Sigma-Aldrich, United States) solution (100 μL) was added to each well and the plates were incubated in the dark at 25°C for 30 min covering with lid and aluminum foil. The absorbance was measured at 517 nm in a microplate reader. Ascorbic acid (Merck, Germany) and butylated hydroxytoluene (BHT) (Merck, India) were used as standards. Each sample was screened in triplicate.

The ABTS radical scavenging assay was performed according to a previously published protocol (Zhen et al., 2016) with some modifications. A 7 mM stock solution of ABTS (Sigma-Aldrich, United States) was prepared by co-dissolving ABTS and potassium persulfate and keeping the resulting mixture for 12–16 h in the dark to form stable radical cations (ABTS^+^). The solution was diluted with ethanol (EtOH) to obtain an absorbance of 0.706 at 734 nm. Endophytic fungal extracts were dissolved in EtOH to prepare stock solutions of 1 mg/mL. The stock solutions were serially diluted into the 96-well plate with EtOH to obtain a range of concentrations from 1,000 to 3.90 μg/mL. The diluted ABTS solution (100 μL) was added to each well and the contents of each well was mixed thoroughly. Decolorisation of the mixture indicated the presence of antioxidant activity in the samples. The absorbance was measured at 734 nm within 1–10 min using a microplate reader. Each sample was screened in triplicate. Ascorbic acid, butylated hydroxytoluene (BHT) and quercetin were used as standards. The percentage of radical scavenging activity of the endophytic fungal extracts in both the DPPH and ABTS assays was calculated as follows;

$$\begin{array}{rcl} \mathrm{Scavenging}\% & = & [({\mathrm{Abs}}_{\mathrm{control}}-{\;\mathrm{Abs}}_{\mathrm{sample}})/{\mathrm{Abs}}_{\mathrm{control}}]\;\times 100\\ {\mathrm{Abs}}_{\mathrm{control}} & = & \mathrm{Only}\;\mathrm{DPPH}\;\mathrm{or}\;\mathrm{ABTS}\;\mathrm{solution}\\ {\mathrm{ABS}}_{\mathrm{sample}} & = & \mathrm{sample}\;\left(\mathrm{extract}\;\mathrm{or}\;\mathrm{standard}\right)\\ & & +\;\mathrm{DPPH}\;\mathrm{or}\;\mathrm{ABTS}\;\mathrm{solution} \end{array}$$

### Ferric Reducing Antioxidant Power (FRAP) Assay

The FRAP assay was performed as previously described (Benzie and Strain, 1996) with some modification. The FRAP reagent was prepared by mixing 10 mM TPTZ (dissolved in 40 mM HCl), 20 mM FeCl_3_.6H_2_O and 300 mM acetate buffer at a ratio 10:1:1. Each endophytic fungal extract (100 μL) was dissolved in distilled water (3.9 mL) in a screw cap test tube and this was mixed homogenously. Then, the FRAP reagent (2 mL) was added and the tube was inverted for several times, followed by incubation at room temperature for 30 min in the dark. The absorbance was measured in a spectrophotometer at 593 nm using distilled water as a blank. A standard curve was plotted for different concentrations (0–50 μM) of FeSO_4_. This allowed to calculate the Ferric Reducing Antioxidant Power (FRAP) values expressed as the content of Fe(II) in μM/mg of dried extract. Quercetin and butylated hydroxytoluene (BHT) were used as positive controls and the samples were tested in triplicate.

### Total Polyphenol Content

The total polyphenol content of each endophytic fungal extract was evaluated using the Folin-Ciocalteu’s reagent following a standard procedure (Anesini et al., 2008). An aliquot (200 μL) of each extract solution (1 mg/mL) was mixed thoroughly with 2 mL of distilled water and 200 μL of Folin-Ciocalteu’s reagent (Sigma-Aldrich, United States). After 3 min, a 7.5% Na_2_CO_3_ solution (1.6 mL) was added and the mixture was incubated in the dark at room temperature for 90 min. The absorbance was measured in a spectrophotometer at 760 nm using distilled water as a blank. A standard calibration curve was generated using six concentrations of gallic acid (6.25–200 μg/mL) and the total polyphenol content was expressed as gallic acid equivalent (GAE) in μg mg^–1^ of dried fungal extract. The samples were tested in triplicate.

### Total Flavonoid Content

The total flavonoid content in each endophytic fungal extract was determined by a colorimetric assay (Saeed et al., 2012) with some modification. An aliquot (300 μL) of each endophytic fungal extract (1 mg/mL) was mixed with 30% methanol (3.4 mL), 150 μL of a 5% sodium nitrite (NaNO_2_) solution, 150 μL of a 10% aluminum chloride (AlCl_3_) solution and 1 mL of a 1M sodium hydroxide (NaOH) solution. All the solutions were mixed thoroughly. Absorbance was measured in a spectrophotometer at 510 nm using distilled water as a blank. A standard calibration curve was generated using six concentrations (6.25–200 μg/mL) of quercetin, and the flavonoid content was expressed as quercetin equivalents (QE) in μg mg^–1^ of dried fungal extract. The samples were tested in triplicate.

### Sulforhodamine (SRB) Assay for Cytotoxicity

All reagents were prepared according to a standard protocol (Wu et al., 2015). Cells were cultured in 15 mL of DMEM (Lonza BioWhittaker^®^), 10% fetal bovine serum, penicillin-streptomycin solution (Corning) and L-glutamine (Lonza BioWhittaker^®^) in a ratio of 100:10:1:1. They were incubated in 5% CO_2_ at 37°C until 75% confluence (Pass-3) was reached. With the help of a hemocytometer, approximately 2 × 10^4^/mL cells were counted and 190 μL of a cell suspension was distributed to each well of a 96-well plate. The microplate was put in the incubator for 24 h (for the “Day 0” measurement, cells were fixed after 2 h of the incubation period). Subsequently, the microplate was checked under the microscope before further proceeding to ensure adequate cell confluence. The test samples were added to each well at five different concentrations (40, 20, 10, 5, and 2.5 μg/mL) and incubated at 37°C for 72 h. After this period, the cells were fixed using 50 μL of ice cold 10% (w/v) trichloroacetic acid (TCA) and kept at 4°C for 1 h. After that, TCA was discarded; the microplate was washed with tap water and dried for 1 h. A 0.4% sulforhodamine B (SRB) solution (100 μL) was added into each well. After incubation for 1 h, each well was washed with 1% acetic acid and dried. A 10 mM Tris base (pH 10) solution (200 μL) was dispensed into each well. The plate was shaken vigorously for 15 min at 120 rpm with a gyratory shaker. Optical density (OD) of each well was measured at 515 nm using an ELISA plate reader (PerkinElmer; 2030 Multilabel Reader, VICTOR^®^ X5). The samples were tested in triplicate.

### α-Glucosidase Inhibitory Assay

This was performed according to a previous protocol (Rivera-Chaìvez et al., 2015) with some modifications. Endophytic fungal extracts were dissolved in dimethyl sulfoxide (DMSO) to prepare stock solutions at 8 mg/mL. An aliquot (50 μL) of each stock solution was serially 2 × diluted with 100 mM potassium phosphate buffer solution (PBS) (pH = 6.8) in a 96-well plate to obtain a range of concentrations for 4–0.031 mg/mL. Then, 20 μL of an α-glucosidase enzyme (Sigma-Aldrich, United States) solution (0.4 unit/mL) was added into each well. Following incubation at 37°C for 10 min, 10 μL of 4-nitrophenyl α-D-glucopyranoside (pNPG) (Sigma-Aldrich, United States) (5 mM) was added, followed by re-incubation at 37°C for a further 30 min. The reaction was terminated by adding 50 μL of a 0.2 M sodium carbonate (Na_2_CO_3_) solution. The absorbance was measured in a microplate reader at 405 nm. SDW was used as a negative control while acarbose was used as standard. The mode of enzymatic inhibition was determined according to a previously described method (You et al., 2011) with some modifications. Each concentration of endophytic fungal extract (8 μL) was incubated with 10 μL of six concentrations of pNPG (2, 1.6, 1, 0.8, 0.4 and 0.2 mM) into 50 μL of a 100 mM PBS (pH = 6.8) solution at 37°C for 20 min within a 96-well plate. Then, 20 μL of an α-glucosidase enzyme solution (0.4 μ/mL) was added to each well. After thorough mixing, the plates were re-incubated for 10 min at 37°C. Following incubation, the reaction was terminated by adding 50 μL of a 0.2 M sodium carbonate (Na_2_CO_3_) solution to every well. The optical density (OD) was measured at 405 nm using a microplate reader. The initial velocity of the reaction was calculated from the relationship of the OD value with time for different substrate concentrations. K_m_ and V_max_ values were determined from the Lineweaver-Burk and Michaelis-Menten plots. The samples were tested in triplicate.

## Results

### Isolation, Preliminary Screening for Biological Activity and Molecular Identification of Fungal Endophytes

A total of 80 fungal endophytes were isolated in this study from different parts of nine medicinal plants from of the Sundarbans mangrove forest. Among them, 35 were isolated from bark, 20 from leaves, 15 from pneumatophores and 10 from stems. Fifteen fungal strains displayed activity in our preliminary biological screening (Supplementary Table 2). This prompted us to identify and scale up the production of each of these strains in order to test for a wider range of biological activity. A nucleotide BLAST search using ITS sequences revealed the identity of each fungal endophyte and their closest matched taxa were enlisted with accession numbers. Despite many attempts, however, some isolates could only be assigned at a genus level rather than a conspecific level due to a lack of similar and relevant sequences in the INSDC databases (Table 1).

**TABLE 1:** Molecular identification of fungal endophytes isolated from nine plants of the Sundarbans mangrove.

Endophyte code	Host plant	Plant part	Genbank accession number	Fungal genus/species	Accession number of closest matched reference species (Genbank)	Percentage identity
AASF-2	*Avicennia alba*	Bark	MT141138	*Penicillium citrinum*	KF624801.1	99.48%
AMSF-3	*Avicennia marina*	Bark	MT141139	*Aspergillus fumigatus*	KX893912.1	99.52%
BSSF-2	*Bruguiera sexangula*	Bark	MT141140	*Aspergillus oryzae*	MH746006.1	99.36%
BSSF-3	*Bruguiera sexangula*	Bark	MT141141	*Aspergillus* sp.	KY809060.1	99.51%
BSSF-4	*Bruguiera sexangula*	Bark	MT141142	*Aspergillus terreus*	MG250398.1	99.36%
BTSF-1	*Brownlowia tersa*	Stem	MT141143	*Talaromyces* sp.	LT558960.1	98.19%
CDSF-2	*Ceriops decandra*	Bark	MT141144	*Talaromyces* sp.	MH860463.1	97.85%
DTSF-4	*Derris trifoliata*	Stem	MT141145	*Penicillium chrysogenum*	KY352035.1	99.68%
HFSF-1	*Heritiera fomes*	Bark	MT141146	*Trichoderma harzianum*	MG707197.1	99.69%
HFSF-3	*Heritiera fomes*	Bark	MT141147	*Talaromyces* sp.	LT558969.1	98.36%
XGSF-1	*Xylocarpus granatum*	Bark	MT141148	*Talaromyces* sp.	KU556510.1	98.51%
XGSF-2	*Xylocarpus granatum*	Bark	MT141149	*Aspergillus terreus*	KC119206.1	99.53%
XMSF-1	*Xylocarpus moluccensis*	Bark	MT141150	*Talaromyces* sp.	KP851981.1	86.94%
XMTSF-1	*Xylocarpus moluccensis*	Pneumatophores	MT141151	*Penicillium verruculosum*	KC698959.1	98.95%
XMTSF-3	*Xylocarpus moluccensis*	Pneumatophores	MT141152	*Aspergillus fumigatus*	MK453215.1	100.00%

### Antimicrobial Activity

Most of the endophytic fungal extracts exhibited antimicrobial activity with MICs in the range of 0.18–3 mg/mL (Tables 2–4). The highest activity (0.18 mg/mL) was observed for *A. fumigatus* (AMSF-3) and *P. verruculosum* (XMTSF-1) on *K. rhizophila*, and for *Talaromyces* sp. (XMSF-1) *on C. albicans*. Against Gram positive bacteria, *A. fumigatus* [AMSF-3] and *A. terreus* [XGSF-2] showed good activity against the test strains with doses ≤1.5 mg/mL. Few isolates showed a bactericidal effect at concentrations under 3 mg/mL. This was observed only or *A. fumigatus* (AMSF-3) and *A. terreus* (XGSF-2) on the biofilm-producing model bacterium *S. aureus* ATCC 6538, for *T. harzianum* (HFSF-1), and *Talaromyces* sp. (HFSF-3) on *B. subtilis*, and for *A. fumigatus* (AMSF-3), *T. harzianum* (HFSF-1), *A. terreus* (XGSF-2), *Talaromyces* sp. (XMSF-1) and *P. verruculosum* (XMTSF-1) on *K. rhizophila.* Interestingly, *A. fumigatus* (AMSF-3) and *A. terreus* (XGSF-2) showed activity against *S. aureus* ATCC 6538 with equivalent MIC and bactericidal concentration values of 0.75 mg/mL. Against *E. coli*, the best activity was observed for *A. fumigatus* (AMSF-3), *A. terreus* (XGSF-2) and *A. fumigatus* (XMTSF-3) with MIC values of 1.50 mg/mL (no bactericidal effect was observed under 3 mg/mL). *Talaromyces* sp. (XGSF-1), *A. terreus* (XGSF-2), *Talaromyces* sp. (XMSF-1), *P. verruculosum* (XMTSF-1) and *A. fumigatus* (XMTSF-3) showed activity against *Salmonella* species *sv Abony*, but none of the endophytes were active against *S. enterica sv typhimurium.* Endophytes *A. terreus* (XGSF-2), *Talaromyces* sp. (XMSF-1), *A. fumigatus* (AMSF-3) and *Talaromyces* sp. (CDSF-2) showed fungicidal action on either *C. albicans* or *A. braziliensis* at a concentration of 3 mg/mL.

**TABLE 2:** Activity (MIC and MBC values in mg/mL) of endophytic fungal extracts against Gram-positive bacteria.

Endophyte (code)	*S. aureus* (ATCC 6538)		*S. aureus* (ATCC 29737)		*B. subtilis* (ATCC 6633)		*K. rhizophila* (ATCC 9341)	
	MIC	MBC	MIC	MBC	MIC	MBC	MIC	MBC
*P. citrinum* (AASF-2)	1.50	>3.00	0.75	>3.00	0.75	>3.00	0.75	3.00
*A. fumigatus* (AMSF-3)	0.75	0.75	0.37	>3.00	0.75	>3.00	0.18	0.75
*A. oryzae* (BSSF-2)	–	–	–	–	1.50	>3.00	–	–
*Aspergillus sp*. (BSSF-3)	–	–	–	–	0.75	>3.00	1.50	>3.00
*A. terreus* (BSSF-4)	1.50	>3.00	1.50	>3.00	0.75	>3.00	0.75	>3.00
*Talaromyces* sp. (BTSF-1)	–	–	–	–	0.75	>3.00	1.50	>3.00
*Talaromyces* sp. (CDSF-2)	–	–	–	–	–	–	1.50	>3.00
*P. chrysogenum* (DTSF-4)	–	–	–	–	0.75	>3.00	0.37	>3.00
*T. harzianum* (HFSF-1)	1.50	>3.00	0.37	>3.00	0.37	0.37	0.75	0.75
*Talaromyces* sp. (HFSF-3)	–	–	0.75	>3.00	0.75	0.75	–	–
*Talaromyces* sp. (XGSF-1)	–	–	–	–	–	–	0.37	>3.00
*A. terreus* (XGSF-2)	0.75	0.75	0.75	>3.00	0.75	>3.00	0.37	0.37
*Talaromyces* sp. (XMSF-1)	–	–	–	–	1.50	>3.00	0.37	0.37
*P. verruculosum* (XMTSF-1)	1.50	>3.00	0.37	>3.00	0.75	>3.00	0.18	0.37
*A. fumigatus* (XMTSF-3)	0.75	>3.00	0.75	>3.00	0.37	>3.00	0.75	>3
Azithromycin	0.005	0.02	0.005	0.04	0.01	0.09	0.01	0.02
Amoxicillin	0.005	0.02	0.02	0.37	0.01	0.37	0.005	0.01

*–, no activity.*

**TABLE 3:** Activity (MIC and MBC values in mg/mL) of endophytic fungal extracts against Gram-negative bacteria.

Endophyte (code)	*S. enterica sv typhimurium* (ATCC 14028)		*Salmonella spp.* sv *Abony* (ATCC 6017)		*E.coli* (ATCC 8739)	
	MIC	MBC	MIC	MBC	MIC	MBC
*P. citrinum* (AASF-2)	–	–	–	–	–	–
*A. fumigatus* (AMSF-3)	–	–	–	–	1.50	>3.00
*A. oryzae* (BSSF-2)	–	–	–	–	–	–
*Aspergillus sp*. (BSSF-3)	–	–	–	–	–	–
*A. terreus* (BSSF-4)	–	–	–	–	–	–
*Talaromyces* sp. (BTSF-1)	–	–	–	–	–	–
*Talaromyces* sp. (CDSF-2)	–	–	–	–	–	–
*P. chrysogenum* (DTSF-4)	–	–	–	–	–	–
*T. harzianum* (HFSF-1)	–	–	–	–	3.00	>3.00
*Talaromyces* sp. (HFSF-3)	–	–	–	–	–	–
*Talaromyces* sp. (XGSF-1)	–	–	1.50	>3.00	3.00	>3.00
*A. terreus* (XGSF-2)	–	–	1.50	>3.00	1.50	>3.00
*Talaromyces* sp. (XMSF-1)	–	–	3.00	>3.00	–	–
*P. verruculosum* (XMTSF-1)	–	–	1.50	>3.00	3.00	>3.00
*A. fumigatus* (XMTSF-3)	–	–	3.00	>3.00	1.50	>3.00
Azithromycin	0.005	0.09	0.005	0.04	0.09	0.18
Amoxicillin	0.005	0.37	0.02	0.75	<0.005	<0.005

*–, no activity.*

**TABLE 4:** Activity (MIC and MFC values in mg/mL) of endophytic fungal extracts against fungi.

Endophyte (code)	*C. albicans* (ATCC 10231)		*A. brasiliensis* (ATCC 16404)	
	MIC	MFC	MIC	MFC
*P. citrinum* (AASF-2)	–	–	–	–
*A. fumigatus* (AMSF-3)	–	–	0.75	3.00
*A. oryzae* (BSSF-2)	1.50	>3.00	–	–
*Aspergillus sp*. (BSSF-3)	1.50	>3.00	0.37	>3.00
*A. terreus* (BSSF-4)	–	–	–	–
*Talaromyces* sp. (BTSF-1)	–	–	–	–
*Talaromyces* sp. (CDSF-2)	0.75	>3.00	0.37	3.00
*P. chrysogenum* (DTSF-4)	0.75	>3.00	–	–
*T. harzianum* (HFSF-1)	–	–	–	–
*Talaromyces* sp. (HFSF-3)	–	–	–	–
*Talaromyces* sp. (XGSF-1)	–	–	–	–
*A. terreus* (XGSF-2)	0.75	3.00	–	–
*Talaromyces* sp. (XMSF-1)	0.18	3.00	1.50	>3.00
*P. verruculosum* (XMTSF-1)	–	–	–	–
*A. fumigatus* (XMTSF-3)	–	–	–	–
Nystatin	0.01	0.37	0.75	0.75

*–, no activity.*

### Radical Scavenging Activity

**FIGURE 2:**
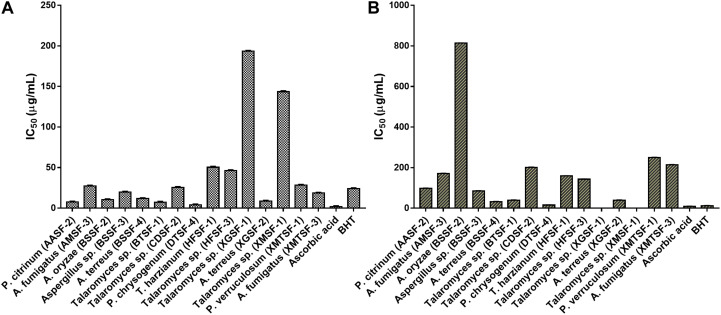
**(A)** ABTS and **(B)** DPPH radical scavenging activity of the endophytic fungal extracts.

The highest DPPH^⋅^ and ABTS^+^ scavenging activity was found for *P. chrysogenum* (DTSF-4) (IC_50_ of 15.92 ± 1.05 and 4.06 ± 1.01 μg/mL, respectively) close to the IC_50_ values obtained for butylated hydroxytoluene (BHT) and ascorbic acid. Radical scavenging activity was also observed for *P. citrinum* (AASF-2), *Aspergillus* sp. (BSSF-3), *A. terreus* (BSSF-4), *Talaromyces* sp. (BTSF-1) and *A. terreus* (XGSF-2) in both assays. Extracts from *A. oryzae* (BSSF-2) *and A. fumigatus* (XMTSF-3) exhibited selective scavenging activity for ABTS^+⋅^ rather than DPPH^⋅^ radicals (Figure 2 and Supplementary Table 3).

### Determination of the FRAP Value and of the Total Polyphenol and Flavonoid Content

**FIGURE 3:**
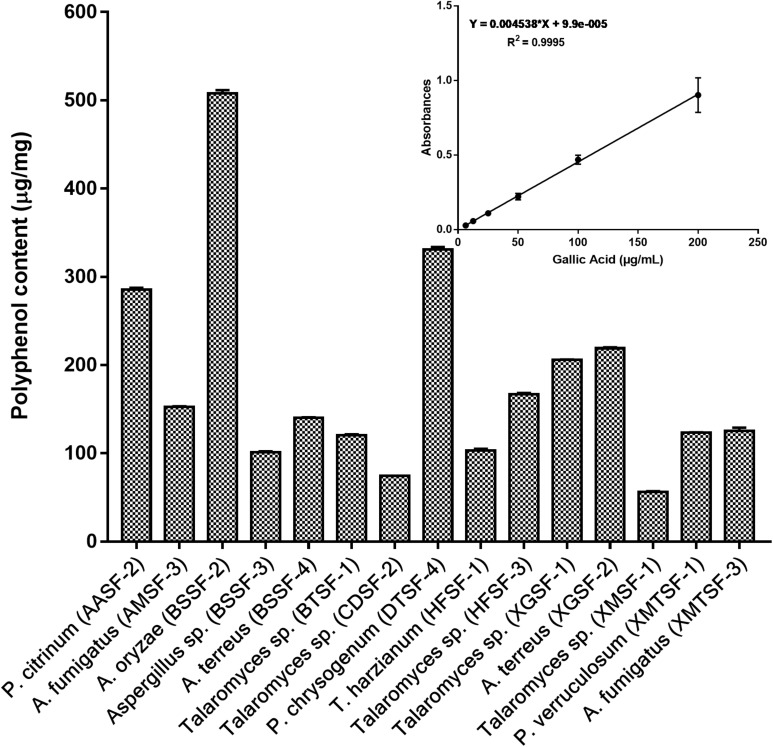
Total polyphenol content within each endophytic fungal extract.

**FIGURE 4:**
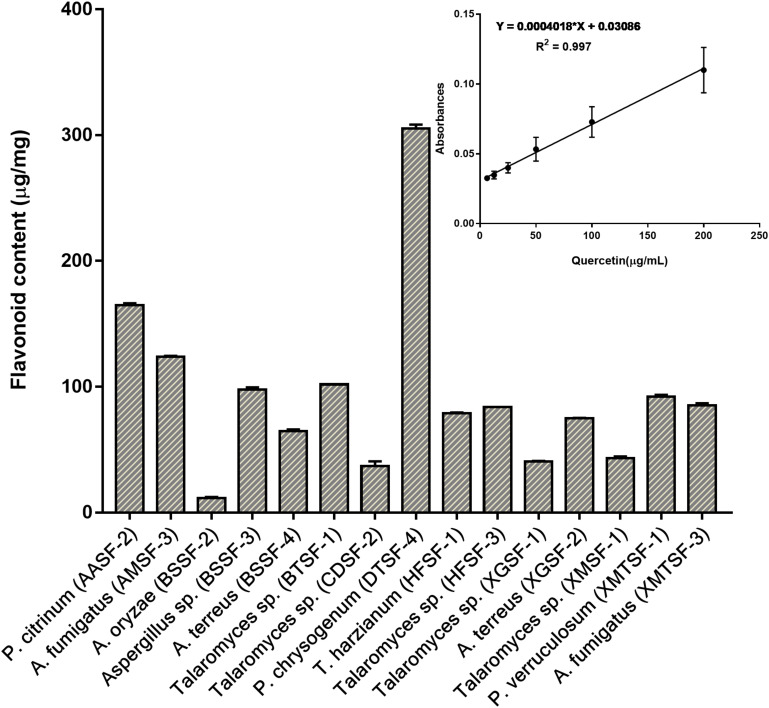
Total flavonoid content within each endophytic fungal extract.

**FIGURE 5:**
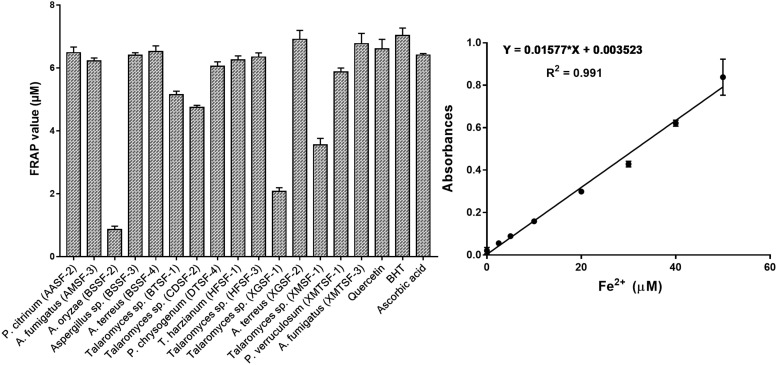
Ferric reducing antioxidant power (FRAP) of each endophytic fungal extract.

The total polyphenolic (Supplementary Table 4 and Figure 3) and flavonoid content (Figure 4) as well as the FRAP values for the endophytic fungal extracts (Figure 5) were calculated from the gallic acid calibration curve, equation: Y = 0.004^∗^X + 9.9e−005(R^2^ = 0.999), quercetin calibration curve, equation: Y = 0.0004^∗^X + 0.03 (R^2^ = 0.997) and Fe^2+^ calibration curve, equation Y = 0.01^∗^X + 0.003 (R^2^ = 0.991). Most of the extracts contained a high amount of total polyphenols and flavonoids and had a high FRAP value. The highest polyphenol content (508.00 μg GAE) was found for the extract of *A. oryzae* (BSSF-2) but this did neither correlate with a high flavonoid content nor a high FRAP value. The highest (and correlated) values for the polyphenolic content, flavonoid content and the FRAP value were observed for *P. chrysogenum* (DTSF-4) (331.00 μg GAE, 305.40 μg QE and 6.05 μM). The isolates *P. citrinum* (AASF-2) and *A. terreus* (XGSF-2) also contained a high quantity of polyphenols (285.30 and 219.10 μg GAE, respectively), among which 164.80 and 74.85 μg QE flavonoid were conserved. The FRAP values of these two fungal extracts (6.49 and 6.91 μM, respectively) were very close to those of quercetin and BHT. Extracts of *Aspergillus* sp. (BSSF-3), *Talaromyces* sp. (BTSF-1), *T. harzianum* (HFSF-1), *P. verruculosum* (XMTSF-1) and *A. fumigatus* (XMTSF-3) also displayed high FRAP values (6.40, 5.15, 6.26, 5.87, and 6.77 μM, respectively).

### Cytotoxic Activity

**FIGURE 6:**
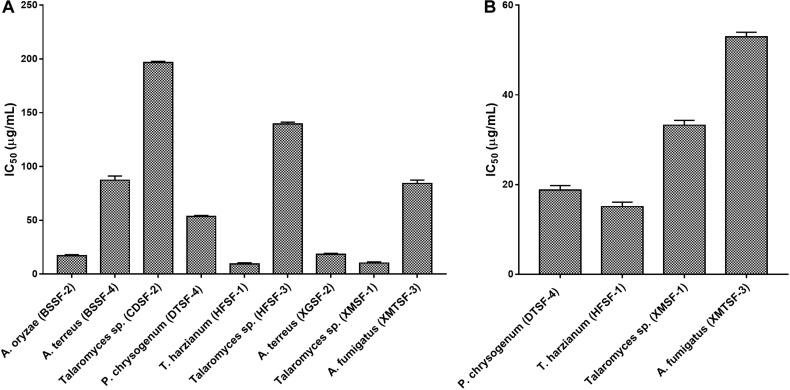
Cytotoxic activity of endophytes against **(A)** MCF-7 and **(B)** SK-LU-1 cell line.

The highest cytotoxic activity on MCF-7 and SK-LU-1 cells was found for *T. harzianum* (HFSF-1) (IC_50_ values of 15.09 ± 1.12 and 9.30 ± 1.00 μg/mL, respectively). *Talaromyces* sp. (XMSF-1) demonstrated noteworthy activity on MCF-7 cell (IC_50_ = 10.29 ± 1.02 μg/mL) but only moderate activity on SK-LU-1 cell. On the other hand, *P. chrysogenum* (DTSF-4) showed pronounced activity on the SK-LU-1 cell line (IC_50_ value of 18.78 ± 1.00 μg/mL) but moderate activity on the MCF-7 cell line. Other endophytes, except for *A. oryzae* (BSSF-2), only showed either week or no activity on our two cell lines. The extract from *A. oryzae* (BSSF-2) was uniquely active on MCF-7 cells with an IC_50_ value of 17.09 ± 1.00 μg/mL, but was inactive on SK-LU-1 cells (Figure 6 and Supplementary Table 5).

### α-Glucosidase Inhibitory Activity

**FIGURE 7:**
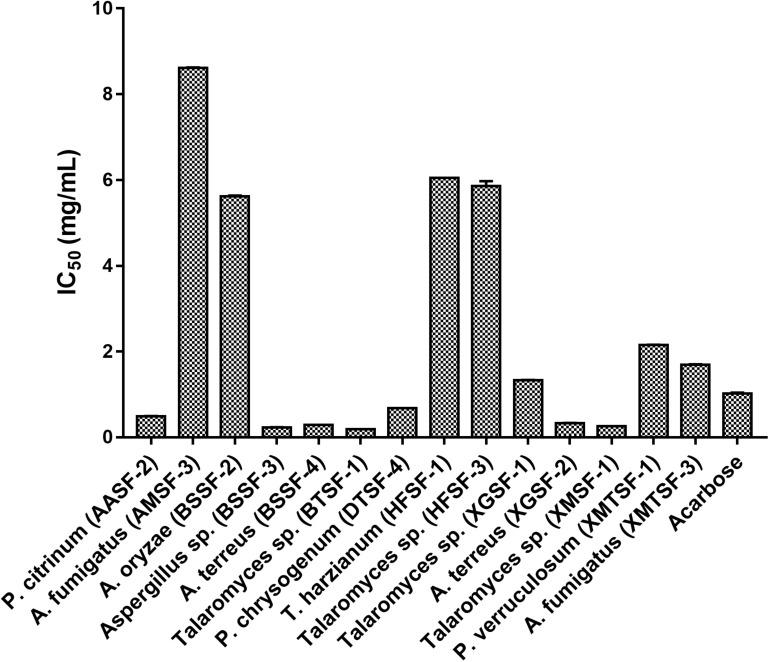
α-Glucosidase inhibitory activity of endophytic fungal extracts.

Several fungal endophytes showed strong α-glucosidase inhibitory activity with *P. citrinum* (AASF-2) (IC_50_ = 0.495 mg/mL), *Aspergillus sp.* (BSSF-3) (IC_50_ = 0.233 mg/mL), *A. terreus* (BSSF-4) (IC_50_ = 0.293 mg/mL), *Talaromyces* sp. (BTSF-1) (IC_50_ = 0.188 mg/mL), *P. chrysogenum* (DTSF-4) (IC_50_ = 0.679 mg/mL), *A. terreus* (XGSF-2) (IC_50_ = 0.333 mg/mL) and *Talaromyces* sp. (XMSF-1) (IC_50_ = 0.258 mg/mL) showing better activity than acarbose (Figure 7 and Supplementary Table 6). A kinetic study, using Michaelis-Menten and Lineweaver-Burk plots to calculate the Michaelis constants (K_m_) and the maximum reaction rates (V_max_), revealed that the different endophytic fungal extracts could inhibit α-glucosidase either non-competitively (*P. citrinum* (AASF-2) and *A. terreus* (XGSF-2)), uncompetitively (*A. fumigatus* (AMSF-3) and *A. terreus* (BSSF-4)) or *via* a mixed inhibition mode [*Talaromyces* sp. (BTSF-1) and *Aspergillus* sp. (BSSF-3)] (Supplementary Table 7 and Figures 1–5).

**FIGURE 8:**
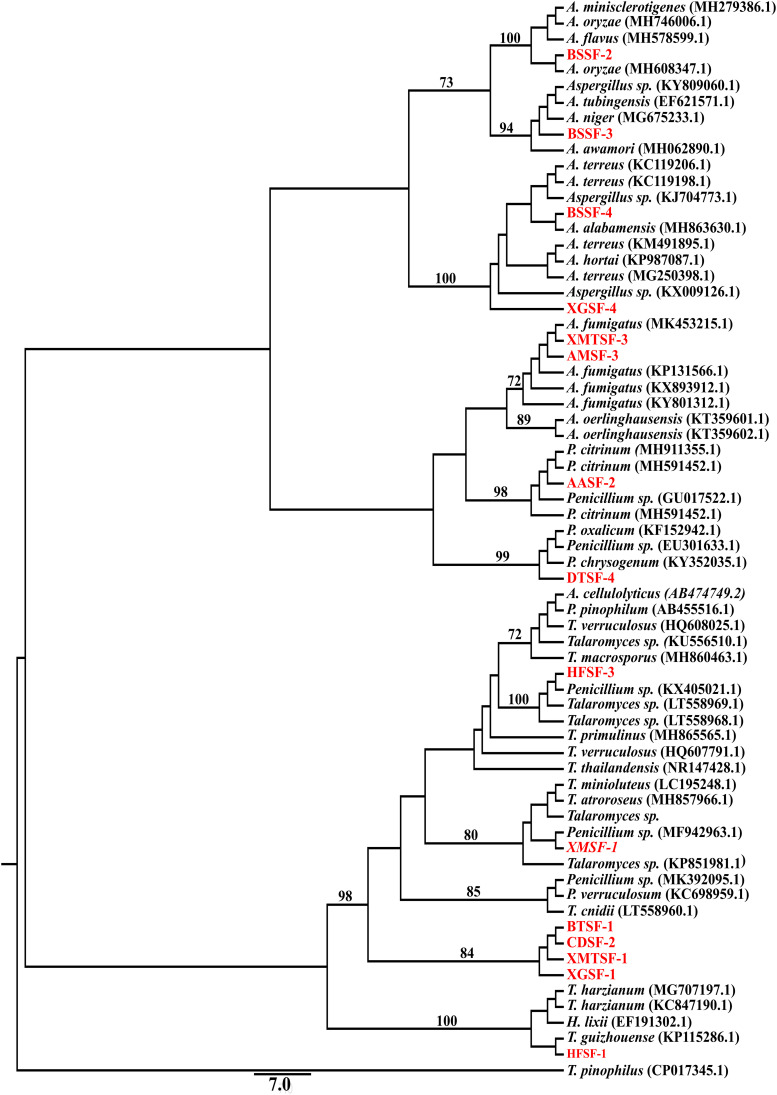
Maximum likelihood tree of the isolated fungal endophytes and their closely associated taxa obtained from GeneBank based on ITS gene sequence. The GeneBank taxa are designated by species name with accession number while our isolates are designated by code name. Only the bootstrap value ≥70% are indicated on the branches. Additional tree information is included within the Supplementary Material.

## Discussion

From the endophytic fungi isolated from nine mangrove plants, fifteen fungal endophytes were selected for further identification. This was done phenotypically and through sequencing of their ITS gene (Olson and Stenlid, 2002; Mishra et al., 2003; Hughes et al., 2013; Raja et al., 2017). This technique is widely used for fungal DNA barcoding (Schoch et al., 2012). We amplified and sequenced the ITS region using ITS5 and ITS4 primers coupled with a heuristic BLAST search.

Fungal endophytes have already been reported in *Avicennia alba* (Lee et al., 2019) and in *A. marina* (Bhimba et al., 2012; Basheer et al., 2018; Bibi et al., 2020). *Penicillium citrinum* was reported in the latter (Liu J. et al., 2015). However, this is the first report of the isolation of *Penicillium citrinum* and *Aspergillus fumigatus* from *A. alba* and *A. marina*, respectively. *Penicillium citrinum* and *Pestalotiopsis foedan* are present in *Bruguiera sexangula* (Xu et al., 2016; Zheng et al., 2019). We report here for the first time the presence of *Aspergillus* species, including *A. oryzae* and *A. terreus* in *Bruguiera sexangula*. Fungal endophytes are also found in *Xylocarpus granatum* (Chokpaiboon et al., 2010; Choodej et al., 2016; Hu et al., 2018; Norphanphoun et al., 2018), *Xylocarpus moluccensis* (Norphanphoun et al., 2018; Wang et al., 2018) and *Heritiera fomes* (Nurunnabi et al., 2018) but all the fungal endophytes we reported in this study are new for these plants. We have identified for the first time some fungal endophytes from *Brownlowia tersa*, *Ceriops decandra*, *Derris trifoliata*.

It is commonly acknowledged that endophytes isolated from mangrove plants have to adapt to a highly competitive environment and that this in turn triggers the production of diverse secondary metabolites that can display great biological potential (Bugni and Ireland, 2004; Cheng et al., 2009). Mangrove fungal metabolites display a range of biological activities (Debbab et al., 2013). The variety of biological effects observed for our endophytic extracts was in good correlation with previous studies which have investigated the antimicrobial, anti-oxidant, anticancer, and antidiabetic potential of fungal endophytes (Chokpaiboon et al., 2010; Buatong et al., 2011; Liu Y. et al., 2015; Choodej et al., 2016; Singh and Kaur, 2016; Nurunnabi et al., 2018). It is interesting to note that high levels of anti-oxidants (i.e., polyphenols/flavonoids) were found within most of our endophytic extracts. Extracts from *A. oryzae* and *A. fumigatus* exhibited selective scavenging activity for ABTS^+⋅^ rather than DPPH^⋅^ radicals. This might be due to the susceptible nature of ABTS^+⋅^ radicals toward both hydrophilic and lipophilic antioxidants whereas the susceptibility of DPPH^⋅^ radicals was only limited to those antioxidants that could be dissolved within the alcoholic assay medium (Cano et al., 2000; Arnao et al., 2001). Extracts of *Aspergillus* sp. (BSSF-3), *Talaromyces* sp. (BTSF-1), *T. harzianum* (HFSF-1), *P. verruculosum* (XMTSF-1) and *A. fumigatus* (XMTSF-3) also displayed high FRAP values.

The fact that our endophytic extracts showed an anti-oxidant effect and that anti-oxidants/polyphenolic compounds are known to have anticancer and antidiabetic activity (Cai et al., 2004; McDougall et al., 2005; De Souza et al., 2010) prompted us to further screen the extracts for anticancer and α-glucosidase inhibitory activity. Extracts from *T. harzianum* (HFSF-1) was found highly active against with IC_50_ values below against the cancer cell lines we used which were below the threshold value (20 μg/mL) defined by the US National Cancer Institute (Wu et al., 2015).

Alpha-glucosidase is an enzyme present in the brush border of the intestine, and which catalyzes the breakdown of carbohydrates for systemic absorption. It is one of the major targets for the treatment of type-2 diabetes mellitus and obesity (Lebovitz, 1997; Kim et al., 2000). In the α-glucosidase inhibitory assay, some endophytic fungal extracts were more potent than the standard drug acarbose and inhibited the enzyme at very low concentrations. This included *Talaromyces* sp. [BTSF-1] (IC_50_ value of 0.188 mg/mL compared to acarbose (IC_50_ value of 1.023 ± 0.02 mg/mL). The presence of polyphenols/flavonoids within this extract may play a role in imparting this inhibitory activity (Xiao et al., 2013).

Overall, we observed that the genus *Aspergillus*, *Penicillium* and *Talaromyces* showed activity against both Gram (+) and Gram (−) bacteria and fungi. They also had anti-oxidant, cytotoxic and α-glucosidase inhibitory activity in good agreement with previous reports (Nicoletti and Trincone, 2016; Wang and Ding, 2018).

## Data Availability Statement

The datasets presented in this study can be found in online repositories. The names of the repository/repositories and accession number(s) can be found in the article/Supplementary Material.

## Author Contributions

MSR and MAI collected the plant specimens. MSR isolated the fungal endophytes, wrote the draft of the manuscript. MSR and SS identified the endophytes. MSR and MAS performed the biological tests, analyzed the results. MAS, MAI, and VS supervised the study design. VS participated in the data analyses, critically reviewed, and finalized the manuscript. All authors contributed to the article and approved the submitted version.

## Conflict of Interest

The authors declare that the research was conducted in the absence of any commercial or financial relationships that could be construed as a potential conflict of interest.
