# Supplementary material for: Molecular Phylogenetics and Biological Potential of Fungal Endophytes From Plants of the Sundarbans Mangrove
Source: Front Microbiol. 2020 Nov 13;11:570855. doi: 10.3389/fmicb.2020.570855 (PMC7691279; doi:10.3389/fmicb.2020.570855)
Supplement: Supplementary file 1 [file Data_Sheet_1.pdf]

Molecular Phylogenetics and Biological Potential of Fungal Endophytes from Plants of the Sundarbans Mangrove

*Md Sohanur Rahaman[1], Md Afjalus Siraj[2], Sabiha Sultana[3], Veronique Seidel[*4], Md Amirul Islam[*1]*

[1]*Pharmacy Discipline, Life Science School, Khulna University, Khulna-9208, Bangladesh.*
[2]*Department of Pharmaceutical Sciences, Daniel K. Inouye College of Pharmacy, University of Hawaii at Hilo, Hilo, HI 96720, USA*
[3]*Agrotechnology Discipline, Life Science School, Khulna University, Khulna-9208, Bangladesh.*
[4]*Natural Products Research Laboratory, Strathclyde Institute of Pharmacy and Biomedical Sciences, University of Strathclyde, Glasgow, UK.*

***Corresponding authors:**
Md Amirul Islam; Pharmacy Discipline, Life Science School, Khulna University, Khulna-9208, Bangladesh, E-mail: maislam26@gmail.com
Veronique Seidel: Strathclyde Institute of Pharmacy and Biomedical Sciences, University of Strathclyde, Glasgow, UK, E-mail: veronique.seidel@strath.ac.uk

Table S1: Plant species collected from the Sundarbans mangrove forest and their GPS (Global Positioning System) sampling coordinates

Table S2: Preliminary bioactivity of fungal endophytes against pathogenic bacteria and fungi

Table S3: Free radical scavenging activity of endophytic fungal extracts

Table S4: Total polyphenol content, flavonoid content and FRAP value of endophytic fungal extract

Table S5: Cytotoxic activity of endophytic fungal extracts

Table S6: α- Glucosidase inhibitory activity (IC_{50}) of endophytic fungal extracts

Table S7: Michaelis constant (K_m) and maximum reaction rate (V_{max}) of α-glucosidase enzyme in the presence of different concentrations of endophytic fungal extracts

Table S8: Phylogenetic tree information

Figure S1: Michaelis-Menten plot and Lineweaver-Burk plot of extract from *Penicillium citrinum* at different substrate (pNPG) concentrations.

Figure S2: Michaelis-Menten plot and Lineweaver-Burk Plot of extract from *Aspergillus fumigatus* (AMSF-3) at different substrate (pNPG) concentrations.

Figure S3: Michaelis-Menten plot and Lineweaver-Burk Plot of extract from *Aspergillus terreus* (BSSF-4) at different substrate (pNPG) concentrations.

Figure S4: Michaelis-Menten plot and Lineweaver-Burk Plot of extract from *Talaromyces sp*. (BTSF-1) at different substrate (pNPG) concentrations.

Figure S5: Michaelis-Menten plot and Lineweaver-Burk Plot of extract from *Aspergillus terreus (*XGSF-2) at different substrate (pNPG) concentrations.

Figure S6: Macroscopic morphology of the pure endophytic fungal isolates after two weeks of incubation on PDA medium.

Table S1: Plant species collected from the Sundarbans mangrove forest and their GPS (Global positioning system) sampling coordinates

Sample number	Plant species (local name)	Latitude *	Longitude *
**1**	*Avicennia marina* (Moricha Baen)	22°13'51.31" N	89° 11' 3.01" E
**2**	*Xylocarpus moluccensis (*Passur)	22°13' 42.13" N	89°11'11.33" E
**3**	*Ceriops decandra* (Goran)	22°13' 42.13" N	89°11'11.33" E
**4**	*Derris trifoliata* (Kali Lota)	22°13' 42.13" N	89°11'11.33" E
**5**	*Heritiera fomes* (Sundri)	22°12'56.56" N	89°12' 4.07" E
**6**	*Xylocarpus granatum* (Dhundul)	22°13'6.31" N	89°13'0.23" E
**7**	*Brownlowia tersa* (Sundri Lota)	22°13'6.31" N	89°13'0.23" E
**8**	*Avicennia alba* (Konok Baen, Sada Baen)	22°8'0.06" N	89°12'34.09" E
**9**	*Bruguiera sexangula* (Kankra)	22°15'44.24" N	89°12'10.04" E

Table S2: Preliminary bioactivity of fungal endophytes against pathogenic bacteria and fungi

		Zoneof inhibition (mm)[a]					
**Endophytes**	**Code**	***B. subtilis***	***S. aureus***	***E. coli***	***S. enterica* sv *typhimurium***	***C. albicans***	***A. brasiliensis***
P. citrinum	AASF-2	16.00 ± 0.57	0.00 ± 0.00	13.83 ± 0.60	0.00 ± 0.00	0.00 ± 0.00	0.00 ± 0.00
A. fumigatus	AMSF-3	14.50 ± 0.28	0.00 ± 0.00	10.83 ± 0.44	0.00 ± 0.00	0.00 ± 0.00	0.00 ± 0.00
A. oryzae	BSSF-2	0.00 ± 0.00	0.00 ± 0.00	12.17 ± 0.44	0.00 ± 0.00	0.00 ± 0.00	0.00 ± 0.00
Aspergillus sp.	BSSF-3	0.00 ± 0.00	8.33 ± 0.88	7.33 + 0.33	0.00 ± 0.00	0.00 ± 0.00	0.00 ± 0.00
A. terreus	BSSF-4	19.83 ± 0.44	13.83 ± 0.60	15.83 ± 0.16	9.50 ± 0.28	0.00 ± 0.00	0.00 ± 0.00
Talaromyces sp.	BTSF-1	12.17 ± 0.60	0.00 ± 0.00	0.00 ± 0.00	0.00 ± 0.00	0.00 ± 0.00	0.00 ± 0.00
Talaromyces sp.	CDSF-2	0.00 ± 0.00	9.00 ± 0.57	8.83 ± 0.44	8.16 ± 0.92	8.50 ± 0.76	0.00 ± 0.00
P. chrysogenum	DTSF-4	11.67 ± 0.88	14.17 ± 0.44	9.83 ± 0.60	9.16 ± 0.44	8.83 ± 0.60	7.50 ± 0.50
T. harzianum	HFSF-1	14.83 ± 0.16	10.33 ± 0.88	9.00 ± 0.57	0.00 ± 0.00	16.00 ± 0.57	0.00 ± 0.00
Talaromyces sp.	HFSF-3	0.00 ± 0.00	10 ± 0.57	9.33 ± 0.33	0.00 ± 0.00	0.00 ± 0.00	0.00 ± 0.00
Talaromyces sp.	XGSF-1	0.00 ± 0.00	8.833 ± 0.44	0.00 ± 0.00	0.00 ± 0.00	0.00 ± 0.00	0.00 ± 0.00
A. terreus	XGSF-2	9.33 ± 0.88	0.00 ± 0.00	0.00 ± 0.00	0.00 ± 0.00	0.00 ± 0.00	0.00 ± 0.00
Talaromyces sp.	XMSF-1	16.00 ± 0.57	15.00 ± 0.57	19.00 ± 0.57	0.00 ± 0.00	0.00 ± 0.00	0.00 ± 0.00
P. verruculosum	XMTSF-1	12.00 ± 0.57	10.67 ± 0.16	11.83 ± 0.44	0.00 ± 0.00	11.50 ± 0.28	0.00 ± 0.00
A. fumigatus	XMTSF-3	10.00 ± 1.15	0.00 ± 0.00	15.5 ± 0.28	0.00 ± 0.00	15.00 ± 0.57	0.00 ± 0.00
Kanamycin		18.33 ± 0.88	12.83 ± 0.60	17 ± 0.57	12.50 ± 0.28	-	-
Ketoconazole		-	-	-	-	19.67 ± 0.88	14.33± 0.33

[a]Data presented as means ± SD; *n* = 3.

Table S3: Free radical scavenging activity of endophytic fungal extracts

**Endophytes**	**Code**	**IC_{50} (µg/mL)[a]**	
		**DPPH radical**	**ABTS radical**
P. citrinum	AASF-2	98.40 ± 1.04	7.56 ± 1.03
A. fumigatus	AMSF-3	171.00 ± 1.07	27.27 ± 1.04
A. oryzae	BSSF-2	813.50 ± 1.16	10.43 ± 1.03
Aspergillus sp.	BSSF-3	84.90 ± 1.10	19.79 ± 1.03
A. terreus	BSSF-4	32.10 ± 1.08	11.81 ± 1.03
Talaromyces sp.	BTSF-1	39.50 ± 1.08	7.35 ± 1.04
Talaromyces sp.	CDSF-2	201.80 ± 1.11	25.50 ± 1.02
P. chrysogenum	DTSF-4	15.92 ± 1.05	4.06 ± 1.01
T. harzianum	HFSF-1	159.00 ± 1.08	50.45 ± 1.09
Talaromyces sp.	HFSF-3	143.00 ± 1.09	46.24 ± 1.03
Talaromyces sp.	XGSF-1	>1000	193.40 ± 1.05
A. terreus	XGSF-2	39.52 ± 1.08	8.59 ± 1.02
Talaromyces sp.	XMSF-1	>1000	143.60 ± 1.03
P. verruculosum	XMTSF-1	250.40 ± 1.04	28.26 ± 1.05
A. fumigatus	XMTSF-3	214.30 ± 1.03	18.52 ± 1.03
BHT (butylated hydroxytoluene)		12.40 ± 1.05	24.10 ± 1.02
Ascorbic acid		9.21 ± 1.01	1.98 ± 1.00

[a]Data presented as means ± SD; *n* = 3.

Table S4: Total polyphenol content, flavonoid content and FRAP value of endophytic fungal extract[a]

Endophytes	Code	Polyphenol content (μg)	Flavonoid content (μg)	FRAP value (μM)
P. citrinum	AASF-2	285.30 ± 2.25	164.80 ± 1.61	6.49 ± 0.17
A. fumigatus	AMSF-3	152.70 ± 0.72	123.80 ± 0.79	6.22 ± 0.09
A. oryzae	BSSF-2	508.00 ± 3.59	11.55 ± 0.86	0.86 ± 0.11
Aspergillus sp.	BSSF-3	101.30 ± 1.11	97.95 ± 1.64	6.40 ± 0.08
A. terreus	BSSF-4	140.40 ± 0.45	64.73 ± 1.36	6.52 ± 0.18
Talaromyces sp.	BTSF-1	120.60 ± 1.15	101.90 ± 0.23	5.15 ± 0.11
Talaromyces sp.	CDSF-2	74.52 ± 0.19	36.93 ± 3.76	4.75 ± 0.06
P. chrysogenum	DTSF-4	331.00 ± 2.96	305.40 ± 2.94	6.05 ± 0.14
T. harzianum	HFSF-1	103.10 ± 2.03	78.95 ± 0.56	6.26 ± 0.12
Talaromyces sp.	HFSF-3	167.10 ± 1.27	83.88 ± 0.01	6.34 ± 0.13
Talaromyces sp.	XGSF-1	206.00 ± 0.36	40.55 ± 0.50	2.08 ± 0.11
A. terreus	XGSF-2	219.10 ± 0.86	74.85 ± 0.33	6.91 ± 0.28
Talaromyces sp.	XMSF-1	56.17 ± 0.87	43.49 ± 1.16	3.55 ± 0.20
P. verruculosum	XMTSF-1	123.70 ± 0.24	92.02 ± 1.52	5.87 ± 0.12
A. fumigatus	XMTSF-3	125.70 ± 3.38	85.22 ± 1.63	6.77 ± 0.32
Quercetin		-	-	6.60 ± 0.30
BHT		-	-	7.03 ± 0.23

[a]Data presented as means ± SD; *n* = 3.

Table S5: Cytotoxic activity of endophytic fungal extracts

Endophytes	Code	IC_{50} (μg/mL)[a]	
		**MCF-7**	**SK-LU-1**
P. citrinum	AASF-2	-	-
A. fumigatus	AMSF-3	-	-
A. oryzae	BSSF-2	17.09 ± 1.00	-
Aspergillus sp.	BSSF-3	-	-
A. terreus	BSSF-4	87.24 ± 3.98	-
Talaromyces sp.	BTSF-1	-	-
Talaromyces sp.	CDSF-2	196.69 ± 1.05	-
P. chrysogenum	DTSF-4	53.50 ± 0.98	18.78 ± 1.00
T. harzianum	HFSF-1	9.30 ± 1.12	15.09 ± 1.00
Talaromyces sp.	HFSF-3	139.61 ± 1.56	-
Talaromyces sp.	XGSF-1	-	-
A. terreus	XGSF-2	18.36 ± 1.00	-
Talaromyces sp.	XMSF-1	10.29 ± 1.02	33.23 ± 1.09
P. verruculosum	XMTSF-1	-	-
A. fumigatus	XMTSF-3	84.28 ± 3.10	52.96 ± 1.01

[a]Data presented as mean ± SD, *n*=3.

Table S6: α- Glucosidase inhibitory activity (IC_{50}) of crude extracts from mangrove fungal endophytes

Endophytes	Codes	IC_{50} (mg/mL)[a]
Penicillium citrinum	AASF-2	0.495 ± 0.003
Aspergillus fumigatus	AMSF-3	8.613 ± 0.015
Aspergillus oryzae	BSSF-2	5.625 ± 0.012
Aspergillus sp.	BSSF-3	0.233 ± 0.002
Aspergillus terreus	BSSF-4	0.293 ± 0.000
Talaromyces sp.	BTSF-1	0.188 ± 0.000
Talaromyces sp.	CDSF-2	21.720 ± 0.003
Penicillium chrysogenum	DTSF-4	0.679 ± 0.000
Trichoderma harzianum	HFSF-1	6.050 ± 0.000
Talaromyces sp.	HFSF-3	5.861 ± 0.110
Talaromyces sp.	XGSF-1	1.334 ± 0.004
Aspergillus terreus	XGSF-2	0.333 ± 0.002
Talaromyces sp.	XMSF-1	0.258 ± 0.000
Penicillium verruculosum	XMTSF-1	2.155 ± 0.007
Aspergillus fumigatus	XMTSF-3	1.696 ± 0.009
Acarbose		1.023 ± 0.019

[a]Data presented as mean ± SD, *n*=3.

Table S7: Michaelis constant (K_m) and maximum reaction rate (V_{max}) of α-glucosidase enzyme in presence of different concentrations of endophytic fungal extracts

Endophytes	Code	Concentration (µg/mL)	K_m (mML^{-1})[a]	V_{max} ($\mu Mmin^{-1}L^{-1}$)[b]	K_i (µg/mL)	Inhibition mode
Penicillium citrinum	AASF-2	0	2.37 ± 0.33	19.86 ± 1.76	550	Noncompetitive inhibition
		20	2.45 ± 0.13	19.60 ± 0.66		
		60	2.45 ± 0.18	17.93 ± 0.86		
		100	2.53 ± 0.22	17.66 ± 1.00		
Aspergillus fumigatus	AMSF-3	0	2.09 ± 0.05	17.22 ± 0.26	103.2*	Uncompetitive inhibition
		100	1.85 ± 0.42	15.14 ± 2.04		
		200	0.62 ± 0.16	4.80 ± 0.46		
		400	0.50 ± 0.02	3.96 ± 0.07		
Aspergillus sp.	BSSF-3	0	1.62 ± 0.06	15.53 ± 0.32	253.1	Mixed inhibition
		20	1.50 ± 0.03	13.96 ± 0.18		
		60	1.42 ± 0.05	13.02 ± 0.26		
		100	1.95 ± 0.11	15.59 ± 0.52		
		150	1.97 ± 0.26	9.98 ± 0.78		
Aspergillus terreus	BSSF-4	0	2.10 ± 0.06	20.15 ± 0.39	102*	Uncompetitive inhibition
		100	1.64 ± 0.04	15.78 ± 0.24		
		200	1.06 ± 0.12	7.32 ± 0.40		
		400	0.82 ± 0.11	5.62 ± 0.32		
Talaromyces sp.	BTSF-1	0	2.60 ± 0.21	21.12 ± 1.13	168.6	Mixed inhibition
		20	1.29 ± 0.05	11.58 ± 0.26		
		60	0.86 ± 0.06	8.52 ± 0.28		
		100	0.56 ± 0.02	6.30 ± 0.10		
Aspergillus terreus	XGSF-2	0	2.02 ± 0.23	18.56 ± 1.31	229.8	Noncompetitive inhibition
		20	2.03 ± 0.35	16.31 ± 1.69		
		60	2.02 ± 0.35	15.72 ± 1.65		
		100	1.91 ± 0.09	11.8 ± 0.33		

[a,b] K_m and V_{max} values presented as mean ± SD, *n*=3.

*Uncompetitive inhibitors did not bind to the enzyme, but to the enzyme-substrate complex

Table S8: Phylogenetic tree information

Sample	Parameter	Custom Information
**1.**	Tree types	Maximum likelihood tree
**2.**	Tree topology search	NNIs
**3.**	Initial tree	BioNJ
**4.**	Model of nucleotides substitution	Custom (010212)
**5.**	Number of taxa	68
**6.**	Log-likelihood	-6661.39
**7.**	Unconstrained likelihood	-5874.42
**8.**	Discrete gamma model-<br>Number of categories:<br>Gamma shape parameter:	Yes<br>4<br>0.428
**9.**	Nucleotides frequencies	- f(A)= 0.20670<br>- f(C)= 0.30420<br>- f(G)= 0.28090<br>- f(T)= 0.20820
**10.**	GTR relative rate parameters	A <-> C 1.69765<br>A <-> G 3.78877<br>A <-> T 1.69765<br>C <-> G 1.00000<br>C <-> T 3.78877<br>G <-> T 1.00000

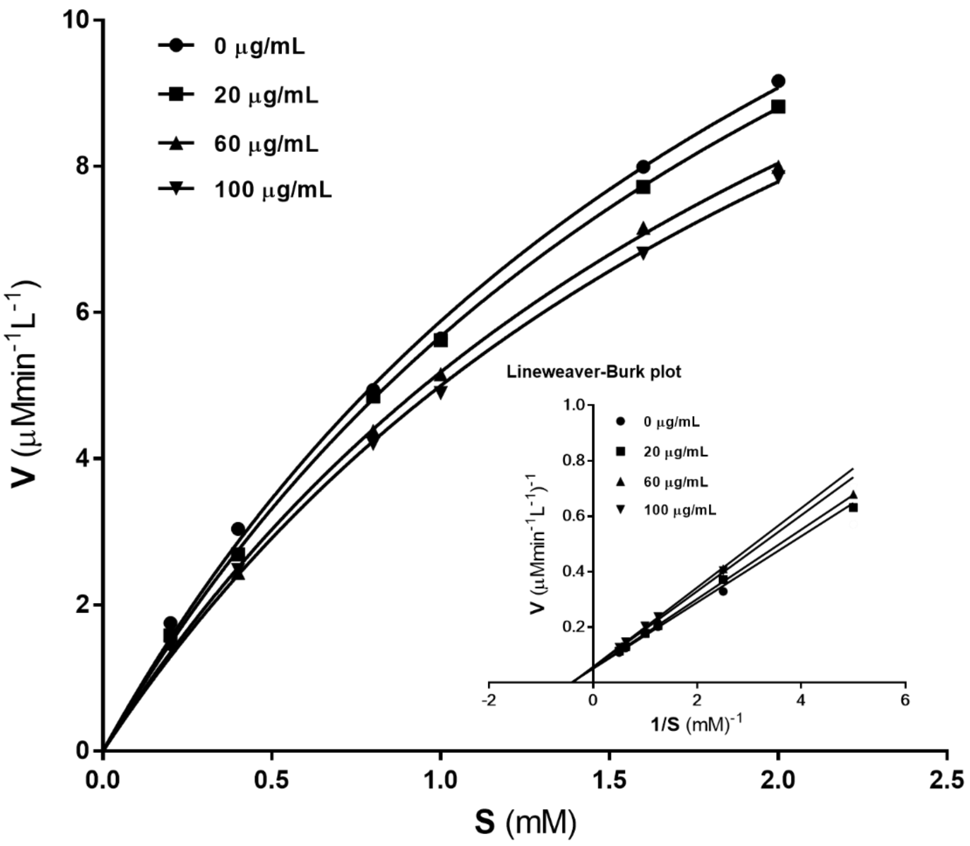

Figure S1: Michaelis-Menten plot and Lineweaver-Burk Plot of extract from *Penicillium citrinum* (AASF-2) at different substrate (pNPG) concentrations.

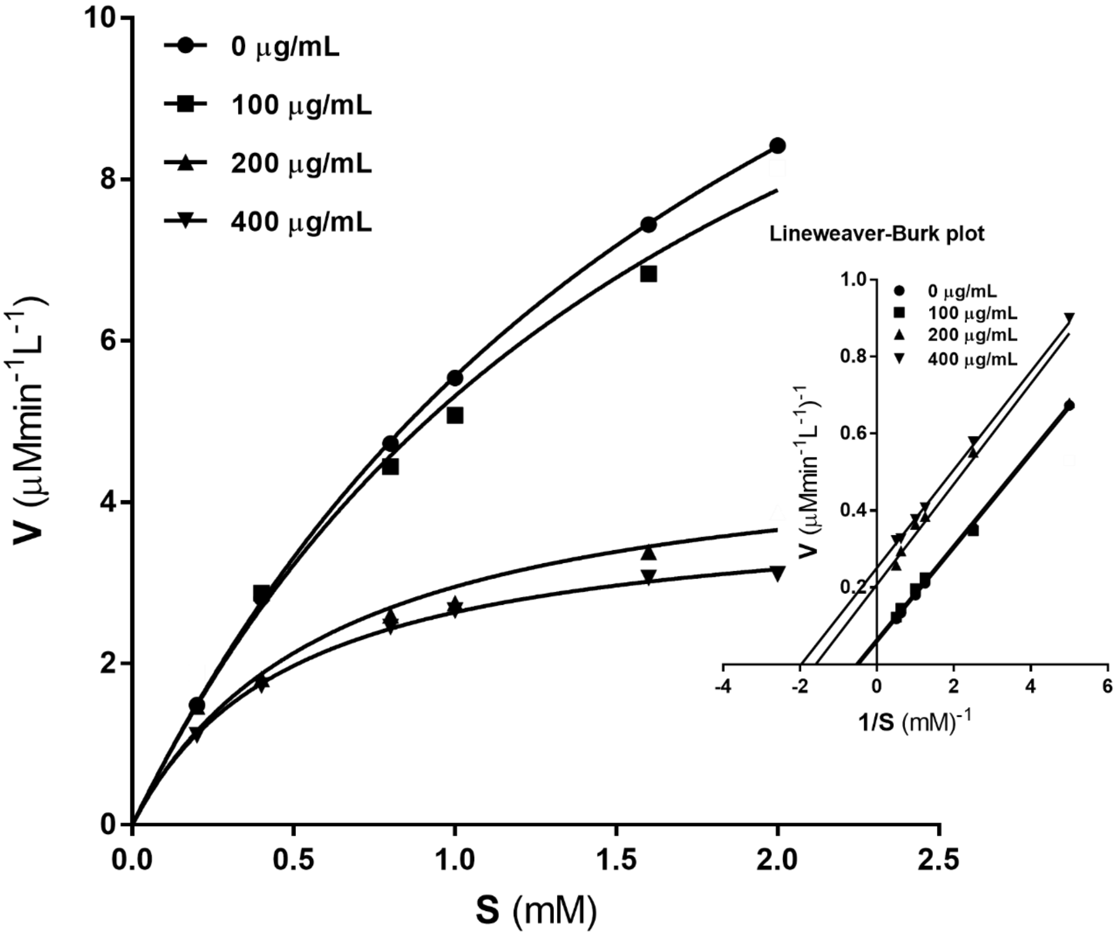

Figure S2: Michaelis-Menten plot and Lineweaver-Burk Plot of extract from *Aspergillus fumigatus* (AMSF-3) at different substrate (pNPG) concentrations.

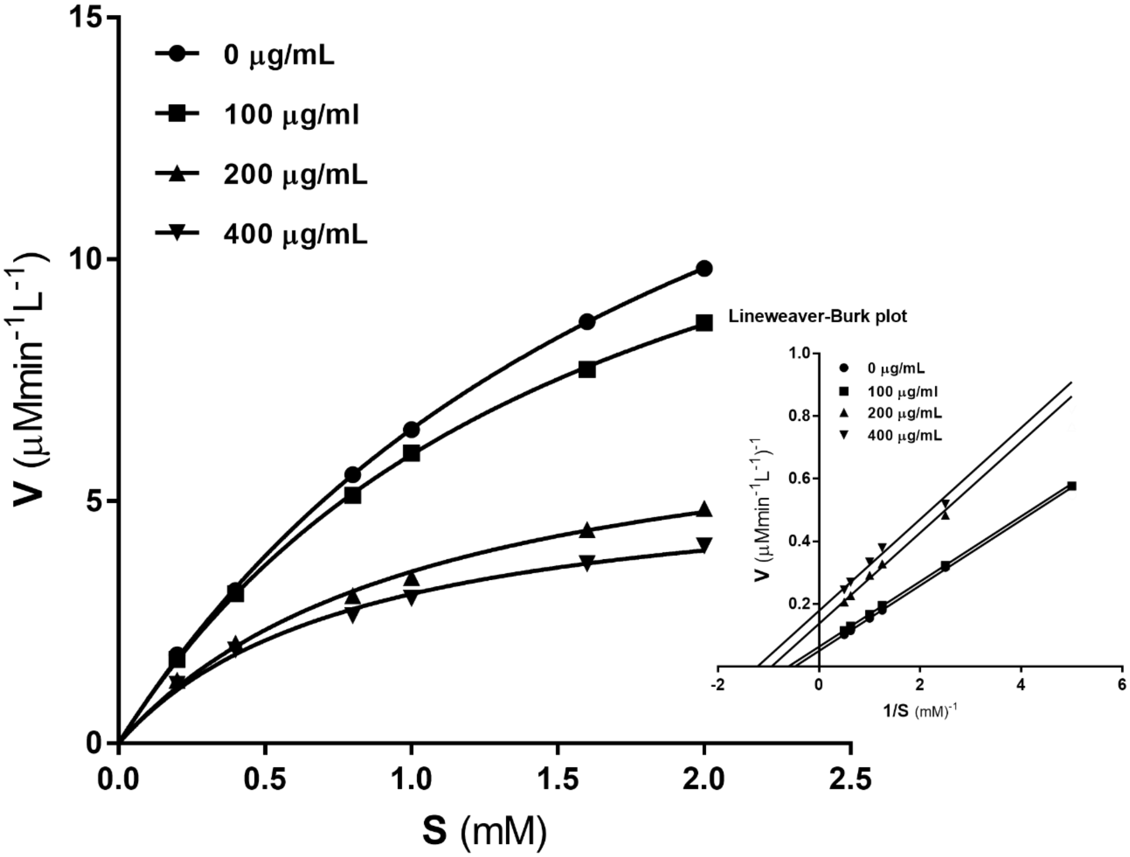

Figure S3: Michaelis-Menten plot and Lineweaver-Burk Plot of extract from *Aspergillus terreus* (BSSF-4) at different substrate (pNPG) concentrations.

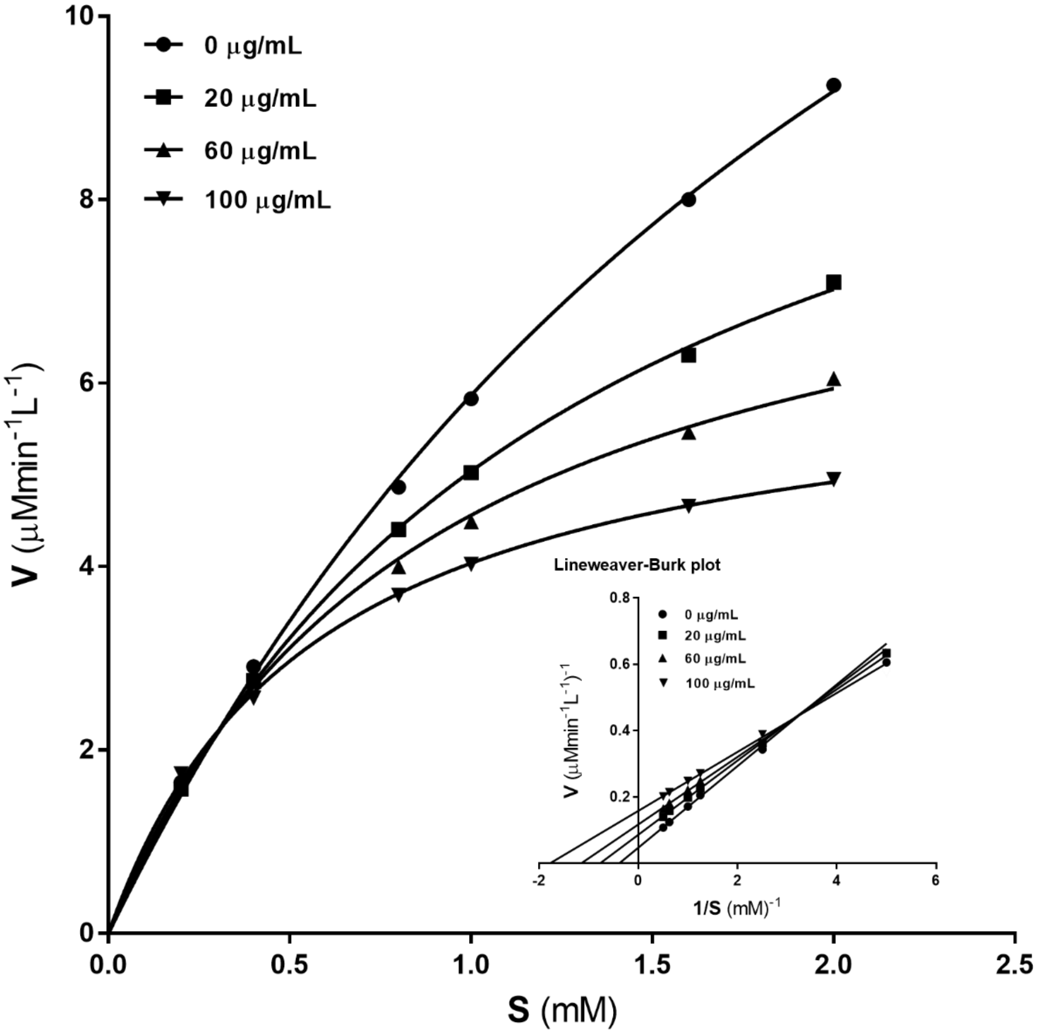

Figure S4: Michaelis-Menten plot and Lineweaver-Burk Plot of extract from *Talaromyces sp.* (BTSF-1) at different substrate (pNPG) concentrations.

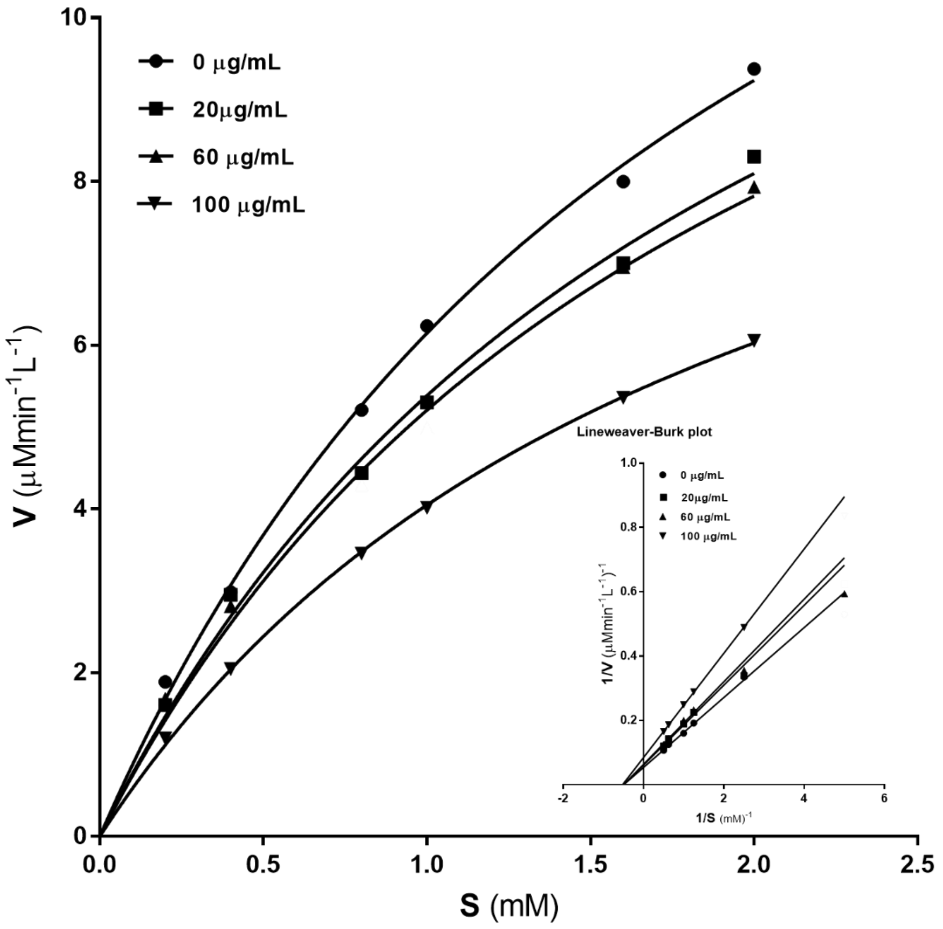

Figure S5: Michaelis-Menten plot and Lineweaver-Burk Plot of extract from *Aspergillus terreus* (XGSF-2) at different substrate (pNPG) concentrations.

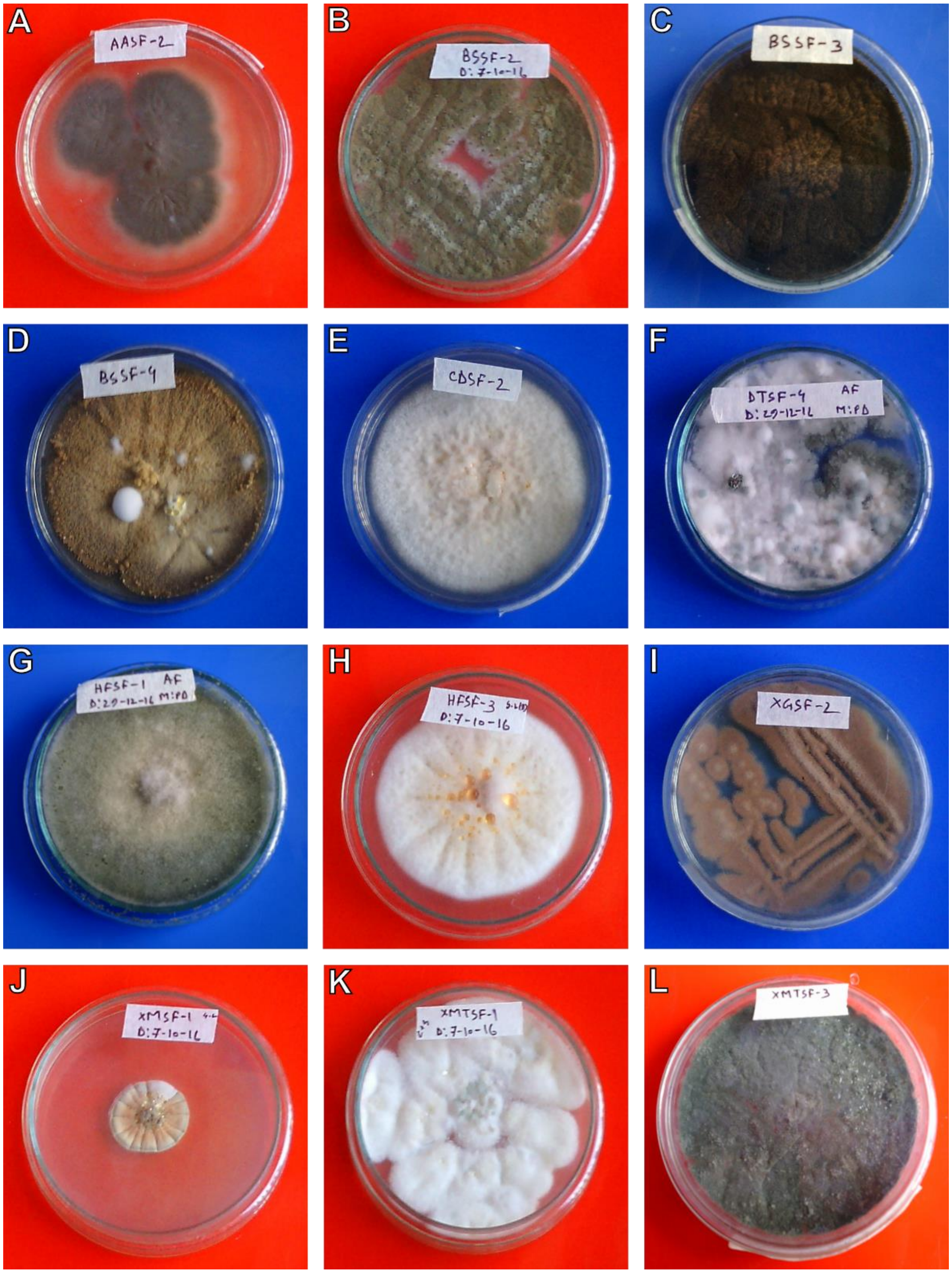

Figure S6: Macroscopic morphology of the pure endophytic fungal isolates after two weeks of incubation on PDA medium.

(A. AASF-2: *Penicillium citrinum*, **B. BSSF-2:** *Aspergillus oryzae,* **C. BSSF-3:** *Aspergillus* sp. **D. BSSF-4:** *Aspergillus terreus*, **E. CDSF-2:** *Talaromyces* sp.**, F. DTSF-4:** *Penicillium chrysogenum*, **G. HFSF-1:** *Trichoderma harzianum*, **H. HFSF-3:** *Talaromyces* sp.**, I. XGSF-2:** *Aspergillus terreus*, **J. XMSF-1:** *Talaromyces* sp.**, K. XMTSF-1:** *Penicillium verruculosum*, **L. XMTSF-3:** *Aspergillus fumigatus***)**
